# Use of Coal Bottom Ash and CaO-CaCl_2_-Activated GGBFS Binder in the Manufacturing of Artificial Fine Aggregates through Cold-Bonded Pelletization

**DOI:** 10.3390/ma13245598

**Published:** 2020-12-08

**Authors:** Dongho Jeon, Woo Sung Yum, Haemin Song, Seyoon Yoon, Younghoon Bae, Jae Eun Oh

**Affiliations:** 1 School of Urban and Environmental Engineering, Ulsan National Institute of Science and Technology (UNIST), UNIST-gil 50, Ulju-gun, Ulsan 44919, Korea; jeondongho@unist.ac.kr (D.J.); wsyum@kict.re.kr (W.S.Y.); haemin93@unist.ac.kr (H.S.); 2 Department of Civil Engineering, Kyonggi University, 154-42, Gwanggyosan-ro, Yeongtong-gu, Suwon-Si, Gyeonggi-do 16227, Korea; yoonseyoon@kyonggi.ac.kr; 3 Advanced Railroad Civil Engineering Division, Korea Railroad Research Institute, 176, Cheoldobangmulgwan-ro, Uiwnag-si, Gyeonggi-do 16105, Korea

**Keywords:** fine aggregate, bottom ash, GGBFS, cold-bonded pelletization, heavy metal leaching

## Abstract

This study investigated the use of coal bottom ash (bottom ash) and CaO-CaCl_2_-activated ground granulated blast furnace slag (GGBFS) binder in the manufacturing of artificial fine aggregates using cold-bonded pelletization. Mixture samples were prepared with varying added contents of bottom ash of varying added contents of bottom ash relative to the weight of the cementless binder (= GGBFS + quicklime (CaO) + calcium chloride (CaCl_2_)). In the system, the added bottom ash was not simply an inert filler but was dissolved at an early stage. As the ionic concentrations of Ca and Si increased due to dissolved bottom ash, calcium silicate hydrate (C-S-H) formed both earlier and at higher levels, which increased the strength of the earlier stages. However, the added bottom ash did not affect the total quantities of main reaction products, C-S-H and hydrocalumite, in later phases (e.g., 28 days), but simply accelerated the binder reaction until it had occurred for 14 days. After considering both the mechanical strength and the pelletizing formability of all the mixtures, the proportion with 40 relative weight of bottom ash was selected for the manufacturing of pilot samples of aggregates. The produced fine aggregates had a water absorption rate of 9.83% and demonstrated a much smaller amount of heavy metal leaching than the raw bottom ash.

## 1. Introduction

Concrete is an artificial material that is widely used for building infrastructure in civil engineering [1] and it is estimated that around 30 billion tons of concrete are produced worldwide every year [2]. However, concrete production requires large quantities of natural fine and coarse aggregates, as about 70% of the total volume of concrete is composed these of aggregates. Therefore, natural aggregates, such as river and sea sand, are constantly being excavated, which destroys the natural environment and depletes natural resources. As interest in environmental conservation grows, many studies have been conducted with the goal of replacing natural concrete aggregates with either recycled concrete aggregates [3] or waste materials [4,5].

Coal bottom ash (bottom ash) is an incombustible residue waste collected from coal-fired power plants [6]. In the Republic of Korea (South Korea), coal fly ash (fly ash), which is also an industrial by-product of coal-fired power plants, has been sold as either a mineral admixture for concrete or as a raw material for Portland cement manufacturing for USD 20−25 per ton, while bottom ash is mostly disposed of in landfills or in ash ponds at a disposal cost of USD 10−20 per ton. Additionally, as bottom ash contains toxic heavy metals, its disposal can cause serious environmental pollution [7].

Previous studies have suggested that bottom ash can be used as a mineral admixture for concrete after (1) grinding to increase its pozzolanic reactivity [8], (2) sieving to increase its fineness [9], (3) the addition of a superplasticizer to reduce its water absorption [10], or (4) putting it in a cement kiln as a raw material [11]. However, it is difficult to use raw bottom ash directly as a mineral admixture for concrete because its use generally decreases the workability of fresh concrete due to the angular grain texture [12] and bottom ash has a lower pozzolanic reactivity than fly ash due to its larger particle size [12].

Conversely, the granulation of bottom ash can be a successful way to increase its utilization rate because it can then be used as an artificial aggregate in concrete production due to its good volumetric stability in hardened concrete [13]. Among the various granulating techniques, the cold-bonded pelletization is a decent candidate as it can shape powdered materials into spherical pellets. By simply spraying water on the powdered materials in a rotating pelletizing disk, powder particles are agglomerated by gravity and friction over time and become spherical pellets [14,15]. Therefore, previous studies have attempted to manufacture artificial aggregates using waste powders such as fly ash, bottom ash, or iron ore fines through the cold-bonded pelletization technique [16,17,18,19].

Additionally, rotary kilns are generally used to manufacture artificial aggregates after the granulation process. However, rotary kilns generally use a sintering process at temperatures near to 1200 °C to form aggregates, which requires the consumption of a great deal of energy [15,20]. It is therefore necessary to develop new methods of producing artificial aggregates that work at relatively low temperatures (e.g., room temperature).

Therefore, this study investigated the use of bottom ash and cementless binder in the manufacturing of artificial fine aggregates using cold-bonded pelletization. This study used the CaO-CaCl_2_-activated ground granulated blast furnace slag (GGBFS) as a cementless binder because it is more economical and environmentally friendly than Portland cement and geopolymer [21,22]. Specifically, the use of geopolymer binder is inappropriate for cold-bonded pelletization because spraying high pH alkaline solutions such as NaOH or water glass on powder materials in pelletizing disks would cause serious safety issues for workers. To this end, mixture samples were prepared using a CaO-CaCl_2_-GGBFS binder with varying additions of bottom ash for strength testing and material analyses, including powder X-ray diffraction (XRD), thermogravimetry (TG), inductively coupled plasma-optical emission spectrometry (ICP-OES), and ion chromatography (IC). After evaluating the mechanical strength and pelletizing formability of the mixtures, one proportion was selected for use in the production of pilot samples of artificial fine aggregates using a disk pelletizer and room temperature curing; water absorption and leaching tests for heavy metals were then conducted for the produced aggregates.

## 2. Materials and Methods

### 2.1. Raw Materials

Bottom ash was collected at the Ha-dong thermal power plant in South Korea. Commercial GGBFS (Chunghae, Korea) was used. Analytical grade CaO and CaCl_2_ (extra pure, 98%) were used as activators.

The oxide and elemental compositions of the raw materials, GGBFS and bottom ash, were determined using an X-ray fluorescence (XRF) spectrometer (S8 Tiger; Bruker, Billerica, MA, USA) and their losses on ignition (LOI) were measured with a thermal analyzer (SDT Q600; TA Instruments, New Castle, DE, USA). Table 1 shows the elemental and oxide compositions of the GGBFS and bottom ash. 

Powder X-ray diffraction (XRD) patterns for the GGBFS and bottom ash were collected using a high-power X-ray diffractometer (D/MAX 2500V/PC; Rigaku, Tokyo, Japan) with a Cu-Kα radiation (λ = 1.5418 Å) for a 2θ scanning range of 8°−60° in 2θ degrees. The obtained XRD patterns were analyzed with the X’pert High Score program [23] using the International Center for Diffraction Data (ICDD) PDF-2 database [24] and the Inorganic Crystal Structure Database (ICSD) [25].

**Figure 1:**
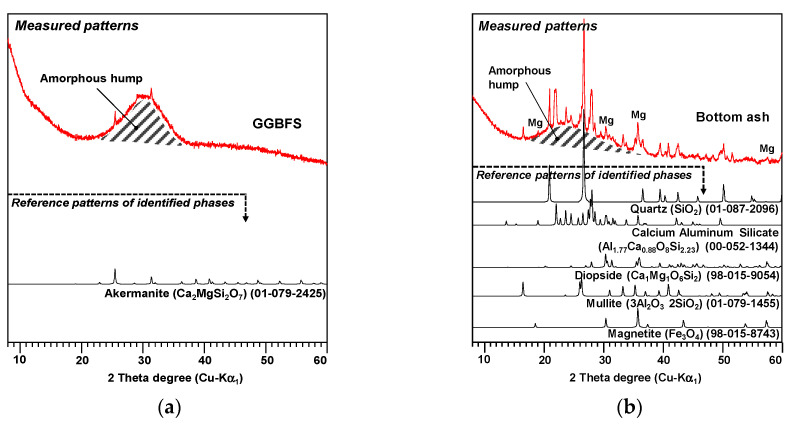
XRD patterns and identified phases of the (**a**) raw GGBFS and (**b**) raw bottom ash. Mg: magnetite.

Figure 1 shows the measured XRD patterns and identified phases of the raw GGBFS and bottom ash. In the mineralogical composition, the GGBFS contained only akermanite (Ca_2_MgSi_2_O_7_, ICSD PDF-2 no. 01-079-2425) while the bottom ash included several crystalline minerals such as quartz (SiO_2_, ICSD PDF-2 no. 01-087-2096), calcium aluminum silicate (Al_1.77_Ca_0.88_O_8_Si_2.23_, ICSD PDF-2 no. 00-052-1344), mullite (3Al_2_O_3_2SiO_2_, ICSD PDF-2 no. 01-079-1455), diopside (Ca_1_Mg_1_O_6_Si_2_, ICDD PDF-2 no. 98-015-9054), and magnetite (Fe_3_O_4_, ICDD PDF-2 no. 98-015-8743). Amorphous humps were observed in both materials and are marked as shaded areas in Figure 1. The GGBFS mostly consisted of amorphous phase while the bottom ash contained a much smaller portion.

**Figure 2:**
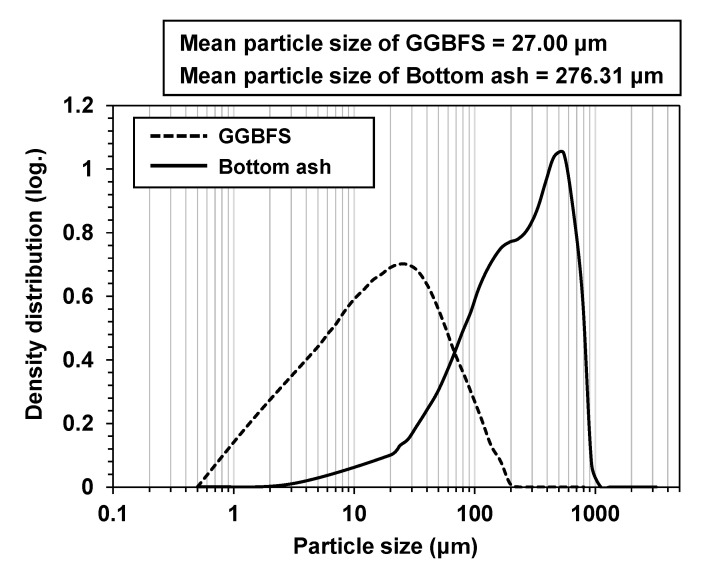
Particle size distributions of raw GGFBS and raw bottom ash with their mean particle sizes.

The particle size distributions of the GGBFS and bottom ash were examined with a laser scattering particle size analyzer (HELOS; Sympatec, Clausthal-Zellerfeld, Germany) (Figure 2). It is worth noting that the mean particle size of the bottom ash (276.31 μm) was approximately 10 times larger than that of the GGBFS (27.00 μm).

**Figure 3:**
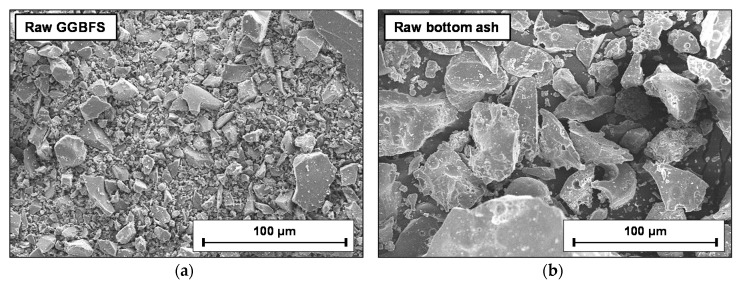
SEM images of (**a**) raw GGBFS and (**b**) raw bottom ash.

The GGBFS and bottom ash were studied using a field emission scanning electron microscope (SEM) (Quanta200; FEI, Eindhoven, The Netherlands) in the secondary electron (SE) mode. The powdered raw materials were placed on double-faced carbon tape with a platinum (Pt) coating. Figure 3 shows SEM SE images of the raw GGBFS and bottom ash.

### 2.2. Sample Preparation and Tests

#### 2.2.1. Mortar Samples

The mixture proportions are given in Table 2. The bottom ash was about 10 times larger in particle size than GGBFS and so it is likely that it acts as a fine aggregate; the mixtures will be referred to as “mortar” in this study although no sand was included in the mixture proportions.

The mortar mixture samples were prepared by adding bottom ash in relative weight ratios of 0, 20, 40, 60, 80, and 100 to the binder weight (GGBFS + CaO + CaCl_2_ = 100) (i.e., B/S0.0, B/S0.2, B/S0.4, B/S0.6, B/S0.8, and B/S1.0 in Table 2, respectively). As the quantity of added bottom ash increased, the relative weight of the binder to the total weight of mixture, which is the binder fraction in mixture (Fr) in Table 2 decreased from 1.0 to 0.5 (e.g., 1.0 for B/S0.0, and 0.5 for B/S1.0). Although the presence of CaCl_2_ could result in the corrosion of the embedded steel bars in concrete, CaCl_2_ was used in the mixture to increase the overall compressive strength of the binder used in this study [21]. The previous study reported that the use of CaCl_2_ in the CaO-activated GGBFS binder system promoted the early dissolution of the amorphous phase of GGBFS considerably [21].

Two values of water-to-binder weight ratio (w/b) were used in the sample preparation: w/b = 2 for diluted samples for ICP-OES and IC and w/b = 0.4 for all other testing samples; the relative weight of water to the total weight of the mixture decreased as the weight of the added bottom ash increased. Thereby, the flowability of the freshly mixed mortar decreased.

The bottom ash was prepared under the surface-dry (SSD) condition before mixing. The GGBFS and CaO powders were dry-mixed with CaCl_2_ and then mixed with varying contents of bottom ash for two minutes in a mechanical mixer. The mixtures were then mixed with de-ionized water for three minutes. The fresh mortars were cast in brass cubic molds (50 × 50 × 50 mm) and then cured for 3, 7, 14, and 28 days. All samples were stored in a humidity chamber at 23 °C with 99% relative humidity for all curing periods.

Compressive strength tests were performed on the triplicate cubic samples for each mixture after 3, 7, 14, and 28 days. After testing, the fractured specimens were collected and finely ground with an agate mortar and pestle. The powdered samples were subjected to a solvent exchange process using isopropyl alcohol to stop further hydration and carbonation for the XRD and TG tests [26].

The XRD patterns for the ground samples were collected and analyzed using the same XRD instrument and analysis program with the database used for analyzing raw materials.

Thermogravimetry (TG) (Q500; TA Instruments, Newcastle, DE, USA) was performed for the ground hardened samples with an alumina pan and nitrogen gas. The heating temperature ranged from room temperature to 1000 °C with a heating rate of 30 °C/min.

Inductively coupled plasma-optical emission spectrometry (ICP-OES) and ion chromatography (IC) were conducted to examine the dissolution behaviors of the GGBFS and bottom ash using a spectrometer (700-ES; Varian, Palo Alto, CA, USA) and a reagent-free ion chromatography system (ICS-3000; Thermo Scientific, Waltham, MA, USA), respectively. In this study, the target elements were calcium (Ca), silicon (Si), aluminum (Al), magnesium (Mg), sulfur (S), and iron (Fe) because the raw materials, GGBFS and bottom ash, were mainly composed of these elements. This was determined by XRF (Table 1).

The sample preparation for ICP-OES and IC analyses was conducted with a water-to-binder weight ratio of 2.0 (w/b = 2.0) at 23 °C for all diluted samples as mentioned earlier. Two hundred grams of water and 100 g of the binder were mixed and then the quantity of bottom ash was increased in increments of 20 g from 0 to 100 g. It is important to note that bottom ash was not included in the binder weight and the bottom ash in SSD was used. Then, the diluted mixtures were consistently agitated using a magnetic stirrer for 24 h at room temperature. After agitation, the filtrated liquid phases from the mixtures were tested for ICP-OES and IC measurement [27].

#### 2.2.2. Aggregate Samples

Pilot samples of the artificial fine aggregates were manufactured for all mixture proportions in Table 2 through cold-bonded pelletization using a disk pelletizer. First, the binder was dry-mixed with varying quantities of bottom ash using the same mixture proportions as in Table 2, except for the fact that the bottom ash was used in a dry state because wet mixing is not necessary for pelletization.

**Figure 4:**
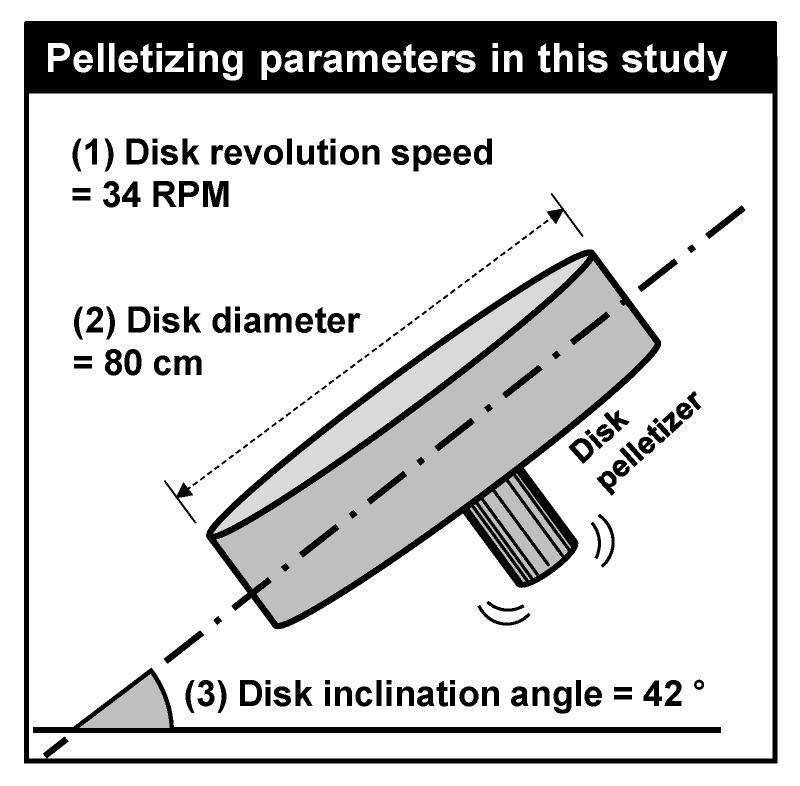
Pelletizing parameters for maximum pelletization efficiency in this study.

The mixed powders were put in a disk pelletizer with a diameter of 80 cm and then pelletized by spraying water. Previous studies noted that disk inclination angle and disk revolution speed affect the efficiency of pelletization [28,29] and after several adjustments were made, the best disk inclination angle and revolution speed were found to be 34 RPM and 42°, respectively, as illustrated in Figure 4.

The freshly pelletized aggregates were cured in a humidity chamber for 28 days at room temperature. Finally, cured aggregates smaller than 4.75 mm were separated via sieving for use as fine aggregates.

After considering the results of the strength test on the mortar samples and the pelletizing formability after the cold-bonded pelletization, the best mixture proportion, B/S0.4, was selected from Table 2 for the production of artificial fine aggregates. The produced aggregates using B/S0.4 were cured in a humidity chamber at 23 °C with 99% relative humidity for 28 days. Aggregates were then used for a water absorption test and a leaching test for heavy metals. Although it is best to conduct the compressive strength and the instrumental analyses for the aggregate sample as well, since some mixture proportions could not be well manufactured into aggregate samples and it is difficult to set the same w/b between the aggregate samples, the identification of reaction products, which is largely influenced by w/b, was conducted only for the samples after the compressive strength test.

The water absorption was measured in the developed 28-day-cured fine aggregate sample according to the ASTM C128-15 [30]. After the fine aggregates of 500 g were submerged in water for 24 h at room temperature, the samples were slowly and equally dried until they reached the SSD condition, as determined by a cone test. The weight of the saturated samples (S) was measured. The samples were dried in an oven at 100 °C for 24 h to determine the oven dry mass (A). The value of the water absorption of the fine aggregates was then calculated following the formula from the ASTM C128-15: Absorption, % = [(S − A)/A] × 100.

The leaching test was performed for both the raw bottom ash and the manufactured fine aggregates [31]. The sample preparation for the leaching test was conducted by following the toxicity characteristic leaching procedure (TCLP) for aggregates of the US Environmental Protection Agency (EPA) to detect concentrations of arsenic (As), cadmium (Cd), barium (Ba), lead (Pb), and chromium (Cr); a solution of pH = 5, which was diluted from 0.5 N acetic acid, was prepared for the extraction medium. The ratio of solution to solid for the samples was set as 20:1 and the diluted samples were agitated with a magnetic stirrer for 18 h. The liquid was tested using ICP-OES after filtrating. Additionally, although the TCLP regulation does not include copper (Cu), zinc (Zn), and nickel (Ni), the concentrations of these elements were also measured using the ICP-OES because a previous study [7] reported that bottom ash contained them in large quantities.

## 3. Results and Discussion

### 3.1. Test Results for Mortar Samples

#### 3.1.1. Compressive Strength Test

**Figure 5:**
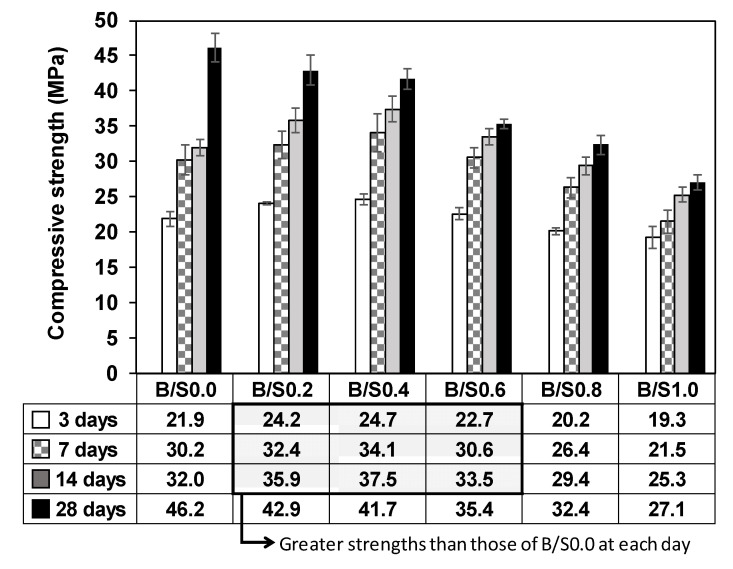
Compressive strength testing results of the mortar mixture samples.

Figure 5 shows the compressive strength testing results for the hardened mortar samples. The averaged values of the three testing results and their standard deviations are indicated in the inset table and as the error bars in Figure 5. All samples showed increasing strength as the curing time increased to 28 days.

The influence of the added bottom ash on strength was dependent on the curing day and the quantity of additional bottom ash. As illustrated in Figure 5, in a range of 20 to 60 relative weight of bottom ash until 14 days (the gray area in the table), the added bottom ash was beneficial in increasing the mortar strength. However, all 28-day strengths were lower than that of B/S0.0 in the same range. For instance, the strength of B/S0.4 at 14 days (37.5 MPa) was ~17% greater than that of the sample without any bottom ash (B/S0.0) (32.0 MPa); however, the strength of B/S0.4 at 28 days (41.7 MPa) was ~10% lower than that of B/S0.0 (46.2 MPa). In fact, all mixture samples regardless of the bottom ash content showed lower mortar strengths at 28 days than B/S0.0 and the 28-day strength decreased as the additional bottom ash increased.

However, it is worth noting that the reduction degrees of 28-day strength were not significant for B/S0.2 and B/S0.4 as compared to those of the other samples; additionally, their strengths were relatively similar to each other despite the difference in the quantity of the bottom ash added, which was twice the ash quantity of B/S0.4 as compared to B/S0.2. As previously stated, USD 10−20 per ton can be obtained as a disposal cost for taking bottom ash from coal-fired power plants; therefore, increasing the bottom ash quantity in the mixture reduces the material cost of manufacturing artificial aggregates in this study. Given the results of strength tests and the possible material costs, the mixture B/S0.4 is likely the best candidate for manufacturing artificial aggregates.

#### 3.1.2. XRD

**Figure 6:**
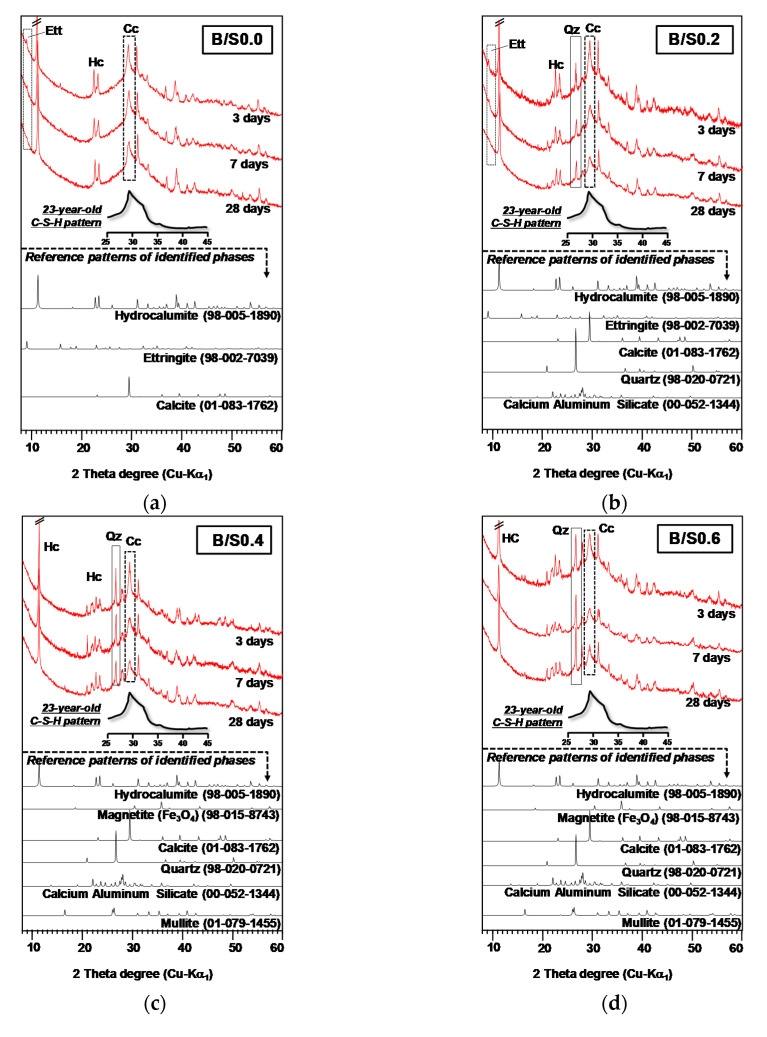

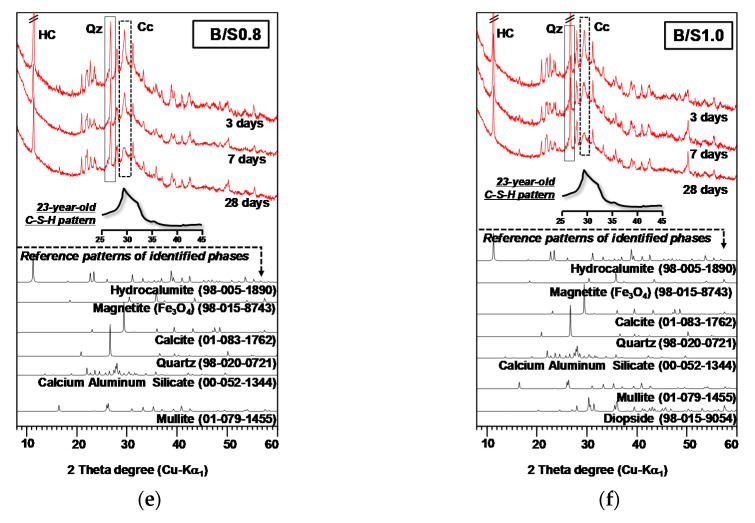
XRD patterns of the hardened mortar mixture samples: (**a**) B/S0.0, (**b**) B/S0.2, (**c**) B/S0.4, (**d**) B/S0.6, (**e**) B/S0.8, and (**f**) B/S1.0; Ett: ettringite, HC: hydrocalumite, Qz: quartz, and Cc: calcite. The XRD pattern of 23-year-old calcium silicate hydrate (C-S-H) was modified from a previous study [32]. The intensities of XRD patterns are modified to have the same baselines between the samples.

Figure 6 presents the XRD patterns of the hardened mortar mixtures at 3, 7, and 28 days.

The sample B/S0.0 showed strong indications of hydrocalumite (Ca_2_Al(OH)_6_Cl·2H_2_O, ICDD PDF-2 no. 98-005-1890) and calcium silicate hydrate (C-S-H) as the main reaction products of the CaO-CaCl_2_-activated GGBFS binder [21]. The formation of C-S-H is seen in the broad peak around 30° according to the XRD pattern of 23-year-old C-S-H from a previous study [32], although the strongest peak of calcite was overlapped with the C-S-H peak around 30° [33]. Additionally, very weak peaks of ettringite (Ca_6_Al_2_(SO_4_)_3_(OH)_2_·26H_2_O, ICDD PDF-2 no.98-002-7039) were identified as a minor reaction product. Although calcite (CaCO_3_, ICSD PDF-2 no. 01-083-1762) was also found in B/S0.0, it was likely not a reaction product of the binder but rather the result of the carbonation of CaO during curing or sample preparation.

In the samples with added bottom ash, hydrocalumite and C-S-H were also identified as the main reaction products. In terms of ettringite, as the weight fraction of bottom ash increased in the mixture, ettringite peaks decreased or disappeared.

In all the samples with added bottom ash, standard bottom ash minerals such ash quartz (SiO_2_, ICDD PDF-2 no. 98-020-0721), mullite (3Al_2_O_3_2SiO_2_, ICSD PDF-2 no. 01-079-1455), calcium aluminum silicate (Al_1.77_Ca_0.88_O_8_Si_2.23_, ICSD PDF-2 no.00-052-1344), diopside (Ca_1_Mg_1_O_6_Si_2_, ICDD PDF-2 no. 98-015-9054), and magnetite (Fe_3_O_4_, ICDD PDF-2 no. 98-015-8743) were found, and as bottom ash content increased, the peak intensities of quartz, mullite, and calcium aluminum silicate became stronger.

#### 3.1.3. TG

**Figure 7:**
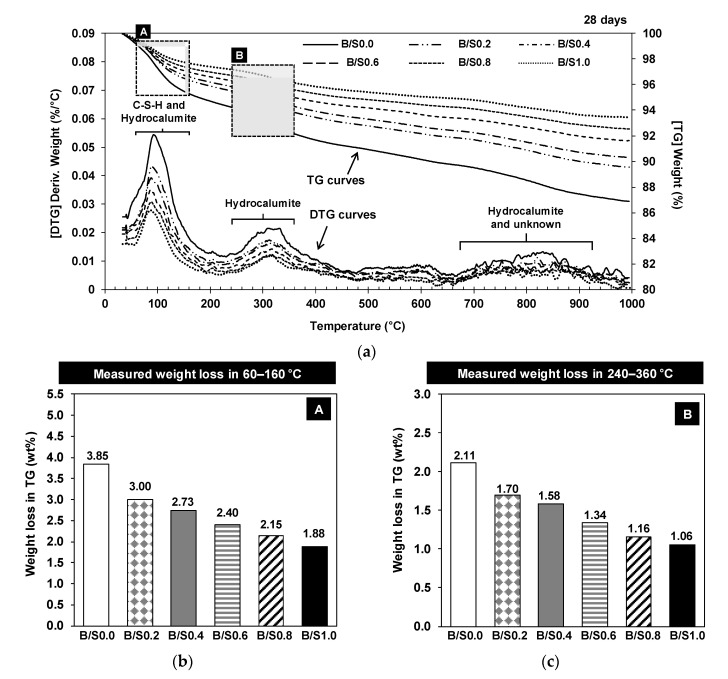
Thermogravimetry (TG) and differential thermogravimetry (DTG) results of the hardened samples at 28 days (**a**) and measured weight losses in 60−160 °C (box A) (**b**) and 240−360 °C (box B) (**c**).

The TG and the differential thermogravimetry (DTG) curves of the hardened mortar mixtures at 28 days are presented in Figure 7. The DTG results confirmed the formation of C-S-H and hydrocalumite, however, the decomposition of calcite was not seen in the temperature range of 650−800 °C [34]. Given that the XRD patterns at 28 days in Figure 7 showed significantly reduced peaks of calcite, it was likely that the calcite amount was not high enough to be detected in TG. Ettringite is known to decompose in the temperature range of 50–200 °C which is indicated by a large DTG peak [35]. In terms of the XRD results, only a small amount of ettringite was found in B/S0.0 and B/S0.2 and no ettringite was found in the other samples. Given that the measured ettringite XRD peaks were too small, it might be difficult to identify the ettringite DTG peaks in B/S0.0 and B/S0.2 due to relatively larger DTG peaks of C-S-H and hydrocalumite. In addition, hydrocalumite was thermally decomposed in three temperature ranges in the TG: (1) 60−160 °C (dehydration), (2) 240−360 °C (dehydroxylation), and (3) above 670 °C (anion decomposition) [26,36].

The dehydrations of C-S-H and hydrocalumite were overlapped around 100 °C in Figure 7 [1,36,37,38], however, because the DTG peaks around 320 °C were only due to the dehydroxylation of hydrocalumite, the weight loss due to the dehydration of C-S-H around 100 °C can be indirectly estimated after assessing the weight loss due to the hydroxylation of hydrocalumite at 240−360 °C [26]. Thus, the influence of added bottom ash on the formation of C-S-H and hydrocalumite can be evaluated.

Figure 7b,c display as if the added bottom ash suppressed the formation of C-S-H and hydrocalumite because the measured weight losses in 60−160 °C and 240−360 °C decreased, respectively, as the added content of bottom ash increased. However, because the binder fraction in mixture (Fr in Table 2) decreased as the bottom ash content increased and C-S-H and hydrocalumite were produced primarily by the binder, the weight loss per binder weight rather than the measured weight loss must be considered in TG.

**Figure 8:**
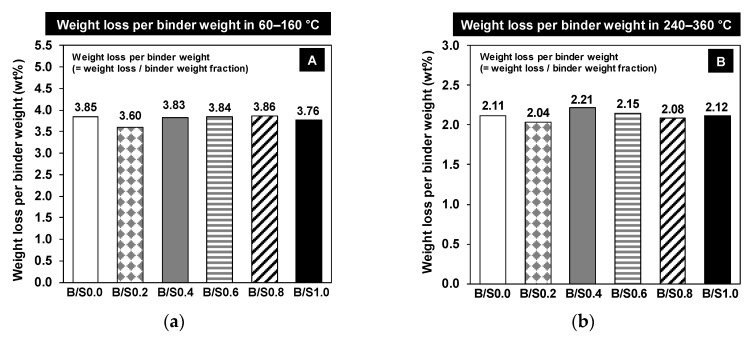
The calculated weight losses per binder weight for 60−160 °C (box A in Figure 7) (**a**) and 240−360 °C (**b**) (box B in Figure 7).

The weight loss per binder weight was calculated by dividing the measured TG weight loss by the binder weight fraction (Fr) in each mixture; Figure 8a,b provide the measured weight losses per binder weight at 60−160 °C and at 240−360 °C, respectively. The figures show that all values were similar in all samples regardless of bottom ash content. This indicates that the addition of bottom ash did not change the amount of C-S-H or the amount of hydrocalumite that was formed over 28 days.

However, it is important to recall that the strength testing results showed that the added bottom ash improved the early strength up to 60 relative weight, although the 28-day strength was not improved. Thus, it can be inferred that the addition of bottom ash may have increased the reaction rate of C-S-H forming in the early days of the study, resulting in early strength improvement, but it did not affect the amount of C-S-H formed up to 28 days nor did it improve the 28-day strength. This is further discussed in Section 3.1.4.

#### 3.1.4. ICP-OES and IC

**Figure 9:**
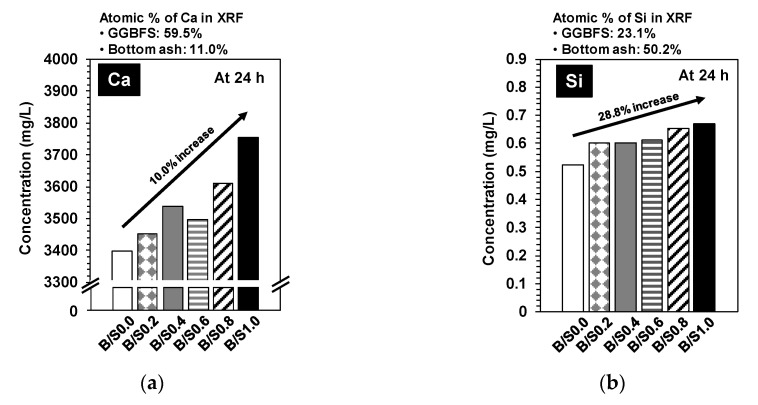

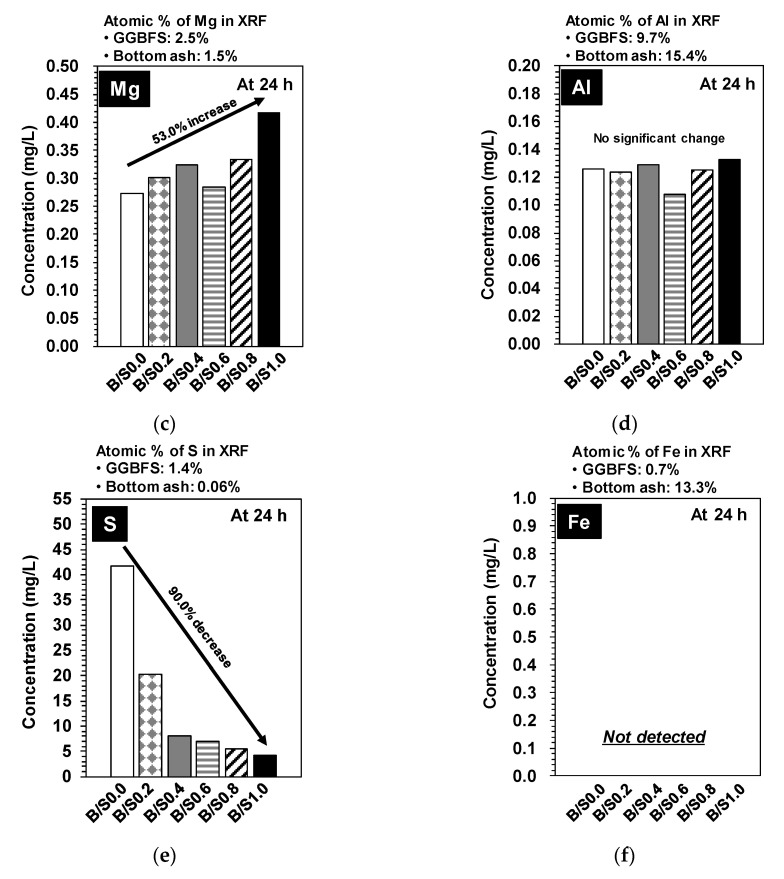
Concentrations of dissolved ions in filtrated liquid from the diluted samples, which were prepared with w/b = 2.0 by agitating for 24 h at 23 °C: (**a**) Ca, (**b**) Si, (**c**) Mg, (**d**) Al, (**e**) S, and (**f**) Fe.

Figure 9 shows the results of the ICP-OES and IC, which illustrate the ionic concentrations of calcium (Ca), silicon (Si), magnesium (Mg), aluminum (Al), sulfur (S), and iron (Fe) in the diluted mixture mortar samples with w/b = 2.0 after 24 h. In Figure 9, the Ca concentrations were significantly larger than those of any other element due to the high solubility of CaCl_2_ [39].

It is worth noting that no Fe ion was detected in any of the samples regardless of the bottom ash content (Figure 9f). The GGBFS had a very small Fe content (0.06 atomic%) and the bottom ash contained considerably more (13.3 atomic%). Thus, no detection implies that Fe existed in an insoluble state in the bottom ash, and, according to the XRD result in Figure 1, most of the Fe in the bottom ash was likely in the form of magnetite (Fe_3_O_4_).

In this study, all elements except Ca were supplied only by GGBFS and bottom ash in the ICP-OES and IC tests. As previously mentioned, all diluted mixture samples had the same weights of binder (GGBFS + CaO + CaCl_2_) and water with w/b = 2.0. The bottom ash was not included in the binder and was incorporated separately into the mixtures and, regardless of the bottom ash content, the weight fractions of GGBFS per water sample were the same in all mixtures. Therefore, if the bottom ash was insoluble or chemically inert, the concentration of the target elements (Ca, Si, Mg, Al, S, and Fe) should not change as the bottom ash content increases. In the results shown in Figure 9, the concentration of Al was not significantly different in all samples, which indicates that Al in the bottom ash was not dissolved in the diluted samples. However, the concentrations of Ca, Si, and Mg notably increased as the bottom ash content increased, and the increased portion of Ca, Si, and Mg were likely due to the dissolution of the additional bottom ash. This indicates that the bottom ash was not entirely inert in the binder reaction but selectively soluble depending on the type of element; that is, Ca, Si, and Mg were likely present in a dissolvable state, while Al was not. Therefore, the improved early strength of the mortar mixture samples in Figure 5 can be explained by the increased concentrations of Ca and Si at an early stage of curing because Ca and Si ions are necessary for forming more C-S-H quickly.

It is also worth noting that sulfur behaved very differently from the other elements; the sulfur concentration was substantially reduced as the bottom ash content increased. The raw GGBFS contained sulfur with 1.4 atomic%, while the raw bottom ash had very little sulfur (0.06 atomic%) in its atomic composition. Thus, unless the added bottom ash affected the dissolution behavior of sulfur in the GGBFS, the sulfur concentration should not have decreased with the addition of bottom ash. Therefore, the results suggest that the presence of the bottom ash inhibited the dissolution of sulfur in the GGBFS, although it is not clear how this inhibition occurred.

### 3.2. Results of Aggregate Production

**Figure 10:**
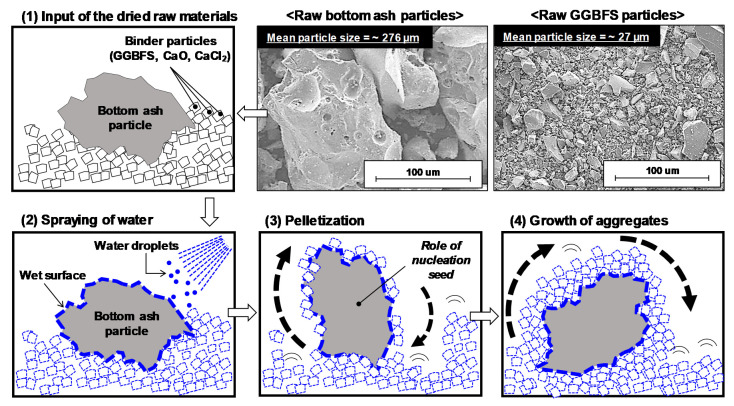
Schematic illustration of the cold-bonded pelletizing process using bottom ash and binder powder (GGBFS + CaO + CaCl_2_).

In this study, the powder was not agglomerated for B/S0.0 and B/S1.0, while the other mixture proportions in Table 2 were suitable for conducting cold-bonded pelletization. In the successful mixtures, the bottom ash particles after water spraying seem to have properly acted as nucleating seeds in the pelletization, as illustrated in Figure 10, due to their rougher surfaces and larger particle sizes as compared to the GGBFS particles in Figure 2 and Figure 3. The agglomeration of aggregate in this study also has price competitiveness compared to the existing artificial aggregate manufacturing technology as it does not require (1) a sintering process, which involves high energy consumption, and (2) additional adhesive chemicals for agglomeration (i.e., water simply acts as a binding agent, as in this study).

**Figure 11:**
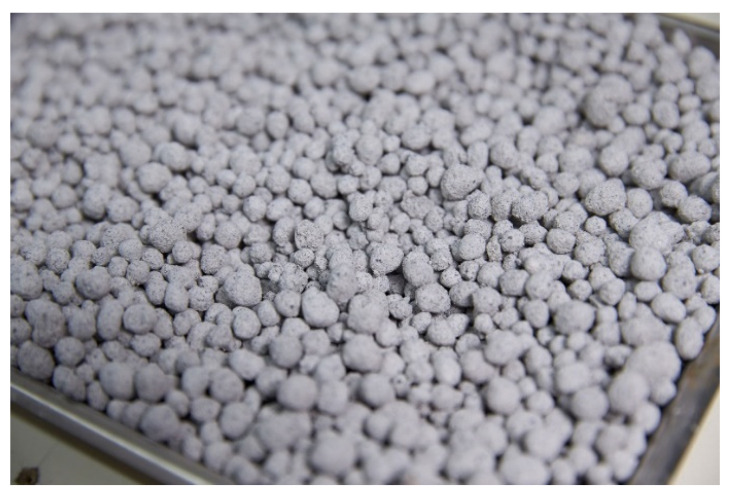
Pilot samples of artificial fine aggregates produced using B/S0.4 after 28 days of curing.

Given the strength testing results of the mortar samples and their pelletizing formability, B/S0.4 was selected as the best mixture proportion in this study. The pilot samples of artificial fine aggregates were therefore produced using B/S0.4 after curing for 28 days, and were relatively spherical, as shown in Figure 11.

**Figure 12:**
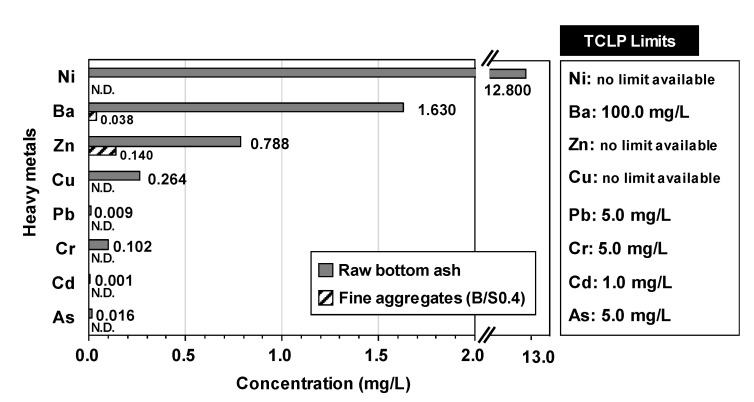
Leaching test results for raw bottom ash and produced fine aggregates (B/S4.0, 28 days). N.D.: not detected. No toxicity characteristic leaching procedure (TCLP) limits are available for Ni, Zn, and Cu.

The result of the water absorption testing for the artificial fine aggregates was 9.83 wt%. According to the KS F 2527 concrete aggregate [40], natural sand should achieve an absolute dry density of 2.5 g/cm^3^ or more and a water absorption of 3.0% or less. However, there is no specification regarding water absorption for artificial fine aggregate manufactured from coal bottom ash. The leaching test (TCLP) results for the fine aggregate sample and the raw bottom ash are shown in Figure 12. Heavy metals such as nickel (Ni), barium (Ba), zinc (Zn), copper (Cu), lead (Pb), chromium (Cr), cadmium (Cd), and arsenic (As) were leached from the raw bottom ash, and the results showed that the concentrations of Ba, Pb, Cr, Cd, and As were lower than the TCLP limits. Although there are no regulation limits on Ni, Cu, and Zn in TCLP, their concentrations were not negligible according to previous studies [7,41]. However, all these metals were leached far less in the produced aggregates (B/S0.4) than in the bottom ash. Specifically, no high concentration was detected for Ni, Cu, Pb, Cr, Cd, or As.

## 4. Conclusions

This study investigated the use of coal bottom ash and CaO-CaCl_2_-activated GGBFS binder in the manufacture of artificial fine aggregates using a cold-bonded pelletization technique.

The adding bottom ash in this study increased early compressive strength before 14 days, although it was not beneficial in improving the 28-day strength. However, when the additional bottom ash was over a certain limit (80 relative weight in this study), the early strength improvement vanished.

The results of the XRD, TG, ICP-OES, and IC tests indicate that the bottom ash was not entirely inert in the binder reaction but was selectively soluble depending on the type of element; that is, Ca, Si, and Mg were likely present in a dissolvable state while Al was not. Additionally, the presence of the bottom ash in the mixture likely suppressed the dissolution of sulfur in the GGBFS.

Although the bottom ash was dissolved to some extent, this did not affect the total amounts of main reaction products (C-S-H and hydrocalumite) formed up to 28 days. However, when a proper amount (up to 60 relative weight) of bottom ash was added, it appears to have increased the reaction rate of forming C-S-H before 14 days, as the concentrations of Ca and Si were increased at the early stage of about 24 h, resulting in increased early strength.

After considering mechanical strength and pelletizing formability, the mixture proportion B/S0.4 was selected for manufacturing artificial fine aggregates. The bottom ash particles seem to have acted as nucleating seeds during the pelletization due to their wet surfaces after water spraying. The water absorption of the artificial fine aggregates was 9.83 wt%. In addition, all target heavy metals were leached far less in the produced aggregates (B/S0.4) than in the bottom ash, and no concentration was detected for Ni, Cu, Pb, Cr, Cd, and As in the produced aggregates.

Thus, this study suggests that the addition of bottom ash to CaO-CaCl_2_-activated GGBFS binder can result in a high-strength artificial fine aggregate for the production of cement mortar bricks and blocks, and can also be used as a concrete coarse aggregate through aggregate size selection.

## Figures and Tables

**Table 1:** Elemental and oxide compositions of ground granulated blast furnace slag (GGBFS) and bottom ash by X-ray fluorescence (XRF).

GGBFS				Bottom Ash			
Element (atomic%)		Oxide (wt %)		Element (atomic%)		Oxide (wt %)	
Ca	59.5	CaO	45.1	Si	50.2	SiO_2_	60.4
Si	23.1	SiO_2_	33.6	Al	15.4	Al_2_O_3_	18.4
Al	9.7	Al_2_O_3_	13.3	Fe	13.3	Fe_2_O_3_	7.4
Mg	2.5	MgO	3.2	Ca	11.0	CaO	6.8
S	1.4	SO_3_	2.1	K	2.0	MgO	1.6
Ti	1.0	TiO_2_	0.8	Ti	1.7	Na_2_O	1.4
Mn	0.8	MnO	0.5	Na	1.5	TiO_2_	1.2
Fe	0.7	K_2_O	0.5	Mg	1.5	K_2_O	1.1
K	0.7	Fe_2_O_3_	0.4	Sr	0.8	MoO_3_	0.4
Na	0.3	Na_2_O	0.3	Mo	0.8	P_2_O_5_	0.3
Sr	0.1	SrO	0.1	Ba	0.7	BaO	0.3
Ba	0.1	BaO	0.1	Nb	0.5	SrO	0.3
Zr	0.09	ZrO_2_	0.1	P	0.3	Tb_4_O_7_	0.1
V	0.03	V_2_O_5_	0.0	Mn	0.2	MnO	0.08
Y	0.02	P_2_O_5_	0.0	Cl	0.06	SO_3_	0.06
P	0.01	Y_2_O_3_	0.0	S	0.03	Cl	0.03
Nb	0.00	Cr_2_O_3_	0.0	Cu	0.03	V_2_O_5_	0.03
LOI (wt %)		0.41%		LOI (wt %)		0.68%	

**Table 2:** Mixture proportions of mortar samples in relative weight.

Label	Binder (b)				Bottom Ash (B)	Binder Fraction in Mixture (Fr) (= S/(S + B))	Water (w)
	GGBFS	CaO	CaCl_2_	Total (S)			
B/S0.0	94	4	2	100	0	1.00	40 (w/b = 0.4)
B/S0.2					20	0.83	
B/S0.4					40	0.71	
B/S0.6					60	0.63	
B/S0.8					80	0.56	
B/S1.0					100	0.50	

Note: For inductively coupled plasma-optical emission spectrometry (ICP-OES) and ion chromatography (IC) testing, the solution samples at w/b = 2.0 were used while the samples at w/b = 0.4 were prepared for all other testing.
